# Mediation of Extracellular Polymeric Substances in Microbial Reduction of Hematite by *Shewanella oneidensis* MR-1

**DOI:** 10.3389/fmicb.2019.00575

**Published:** 2019-03-29

**Authors:** Lei Gao, Xiancai Lu, Huan Liu, Juan Li, Weijie Li, Rongbin Song, Ruiyong Wang, Dongmei Zhang, Junjie Zhu

**Affiliations:** ^1^State Key Laboratory for Mineral Deposits Research, School of Earth Sciences and Engineering, Nanjing University, Nanjing, China; ^2^Key Laboratory of Surficial Geochemistry, Ministry of Education, Nanjing University, Nanjing, China; ^3^School of Chemistry and Chemical Engineering, Nanjing University, Nanjing, China; ^4^School of Life Sciences, Nanjing University, Nanjing, China

**Keywords:** extracellular polymeric substances, extracellular electron transfer, *Shewanella oneidensis* MR-1, hematite, microbial reduction

## Abstract

Extracellular electron transfer (EET) plays a fundamental role in microbial reduction/oxidation of minerals. Extracellular polymeric substances (EPS) surrounding the cells constitute a matrix that separates the cell’s outer membrane from insoluble minerals and environmental fluid. This study investigated the effects of EPS on EET processes during microbial reduction of hematite by the iron-reducing strain *Shewanella oneidensis* MR-1 (MR-1). Electrochemical characterization techniques were employed to determine the influence of EPS components on the redox ability of MR-1. Cells with removed EPS exhibited approximately 30% higher hematite reduction than regular MR-1 cells, and produced a current density of 56 μA cm^-2^, corresponding to 3–4 fold that of regular MR-1. The superior EET of EPS-deprived cells could be attributed to direct contact between outer membrane proteins and hematite surface, as indicated by more redox peaks being detected by cyclic voltammetry and differential pulse voltammetry. The significantly reduced current density of MR-1 cells treated with proteinase K and deoxyribonuclease suggests that the electron transfer capacity across the EPS layer depends mainly on the spatial distribution of specific proteins and electron shuttles. Exopolysaccharides in EPS tend to inhibit electron transfer, however they also favor the attachment of cells onto hematite surfaces. Consistently, the charge transfer resistance of cells lacking EPS was only 116.3 Ω, approximately 44 times lower than that of regular cells (5,139.1 Ω). These findings point to a negative influence of EPS on EET processes for microbial reduction/oxidation of minerals.

## Introduction

Extracellular electron transfer (EET) has been extensively studied in dissimilatory metal-reducing bacteria, and was found to depend on outer membrane protein c-type cytochromes (MtrB, OmcA, MtrC) (Shi et al., 2007) or surface pili (Eggleston et al., 2006; Gorby et al., 2006; Smith et al., 2013). Various electron shuttles (such as flavine) or chelators (such as humic acid) have also been shown to facilitate intracellular and extracellular respiration (Liu et al., 2016; Xiao et al., 2018).

In natural environments, microbes, which commonly appear in the form of multicellular aggregates glued together by extracellular polymeric substances (EPS), are located at oxidation-reduction interfaces (Costerton et al., 1999). EPS appear as an amorphous gel that surrounds the cells (Jiao et al., 2010; Sheng et al., 2010) and accounts for 50–90% of total organic carbon in biofilms (Flemming, 2016). In general, EPS are made of polysaccharides (>90% in weight) as well as proteins, nucleic acids, curli, fimbriae, cellulose, and amyloid formations (Halliwell and Gutteridge, 1990; Bala Subramanian et al., 2010). So far, approximately 20 extracellular and outer membrane proteins have been identified in EPS, but only c-type cytochromes and flavine have been shown to participate in EET processes (Harper et al., 2008; Cao et al., 2011; Harish et al., 2012).

When insoluble minerals act as electron acceptors, the interaction between cells and minerals is constrained by the availability of physical contact (Lovley, 2008; Ouyang et al., 2014; Beblawy et al., 2018). Importantly, EPS are thought to mediate the adhesion of cells to solid surfaces and commonly support biofilm development (Kreft and Wimpenny, 2001; Flemming et al., 2007). Moreover, the electrons must be transported across the inner membrane (MacKenzie et al., 2008), periplasm (Jiao and Newman, 2007), outer membrane (Reguera et al., 2005; Shi et al., 2012, 2016), and EPS layer before reaching the mineral surface. EET processes have been intensively investigated in the last decade (Li et al., 2017; Liu et al., 2017), however, there is no universal mechanisms to assess the importance and influence of EPS in EET. A previous study suggested there was no need to establish an electrical connection with a soluble extracellular electron acceptor that was reduced by microorganisms (Cao et al., 2011). However, for insoluble terminal minerals, direct physical contact with the cell surface is absolutely required, and EPS represent the only such direct physical link (Yundang et al., 2016; Min et al., 2017). Previous studies have regarded the EPS layer as an electronic storage center, and electron “hopping” has been proposed as the most likely mechanism for electron transfer across the EPS layer (Yong et al., 2017). However, polysaccharides, including galactose, glucose, mannose, as well as *N*-acetyl glucosamine, glucuronic acids, and fucose (Lawrence et al., 2016), unavoidably interfere with EET processes (Kouzuma et al., 2010; Kitayama et al., 2017). This dichotomy makes it difficult to evaluate the overall influence of EPS on EET processes and the roles of different EPS components remain unexplored. Further studies are necessary to ascertain the role of EPS in EET between dissimilatory metal-reducing bacteria and metal oxides.

In this study, *Shewanella oneidensis* MR-1, an extensively characterized and widely distributed iron-reducing bacterial strain, was selected to reduce hematite, and the role of EPS was assessed by experimental and electrochemical methods. *Shewanella oneidensis* MR-1 is a gram-negative organism (Oram and Jeuken, 2016) and its respiratory electrons have to cross two membranes before reaching the cell surface, where they come in contact with a solid electron acceptor. Three types of MR-1 cells were prepared: (a) cells whose EPS were removed by physical centrifugation and EDTA-Na_2_ exchange, (b) cells treated *in situ* with protein and DNA-digesting enzymes, and (c) untreated cells. This study investigated the effects of various EPS components on EET between MR-1 cells and hematite using a single three-electrode chamber of a microbial fuel cell (MFC) with different cells on the anode. Based on experimental results of hematite microbial reduction and electrochemical measurements, the EET capacity of different cells as well as the contribution of EPS components to EET processes are discussed.

## Materials and Methods

### Bacterial Culture and EPS Removal

*Shewanella oneidensis* MR-1 (ATCC 700550) was purchased from the ATCC (Manassas, VA, United States). The cells were cultured in tryptic soy broth medium for 48 h at 30°C on a rotary shaker (150 rpm). The medium contained tryptone (17 g L^-1^), soybean meal papain (3 g L^-1^), sodium chloride (5 g L^-1^), potassium hydrogen phosphate (2.5 g L^-1^), and glucose (2.5 g L^-1^), and had a final pH of 7.3 ± 0.2 (Edmonds and Cooney, 1969). Stationary phase cells (OD_600_ = 0.8) were harvested by centrifugation (6,000 ×*g*, 10 min), washed three times with 0.9% (wt vol^-1^) NaCl, and the pellets were resuspended in 0.9% (wt vol^-1^) NaCl for further assays. To remove EPS, the collected cells were treated with 0.5% (wt vol^-1^) EDTA-Na_2_ for 3 h in an ice bath on a rotary shaker (150 rpm). After centrifugation (10,000 ×*g*, 20 min), the cell pellet was resuspended in 0.9% (wt vol^-1^) NaCl and the cell suspensions adjusted to a concentration of 15 mg wet cell mL^-1^. Untreated cell suspensions were denoted as MR-1, whereas cell suspensions whose EPS had been removed were denoted as MR-1-EPS. The supernatants containing EPS (denoted here as EPS) were collected, filtered through 0.45-μm filter membranes, and stored at 4°C (Jorand et al., 1995; Gao et al., 2014).

### Characterization of EPS and Cell Surfaces *in situ*

The amount of EPS extracted from MR-1 cells was determined by calculating the ratio of total organic carbon (TOC) per weight of wet cells. The TOC of EPS was measured by wet combustion using a TOC analyzer (TOC-4100; Shimadzu, Kyoto, Japan).

Exopolysaccharides of different cell suspensions were quantified *in situ* using a periodic acid-Schiff kit (MX594S50-1KT; Sigma-Aldrich, St. Luis, MO, United States). Briefly, 25 μL of cell suspension was added to each well, followed by addition of 175 μL of a 0.5% periodic acid solution prepared in 5% acetic acid (pH 4.0 ± 0.5). The plate was incubated for 30 min at room temperature, after which 100 μL Schiff reagent was added to each well and was made to react for another 120 min. Absorbance at 544 nm was measured with a microplate reader (M200 Pro; Tecan, Männedorf, Switzerland). Dextran from *Leuconostoc* sp. (Mr 50,000, 31389; Fluka, Buchs, Switzerland) was dissolved in pure water and was used as standard (0–1,500 μg mL^-1^).

Exoproteins were assayed using a QuantiPro BCA kit (Sigma-Aldrich) following the manufacturer’s instructions. Bovine serum albumin (BL521A; Biosharp, KeyGen Biotech, Nanjing, China) was used as standard (0–1,000 mg mL^-1^). Absorbance at 562 nm was recorded and protein concentration was determined by comparison to the standard curve.

Zeta potential (mV) and size distribution were determined on a laser particle size analyzer (MS2000; Malvern Instruments, Malvern, United Kingdom) at pH 7.0 in 0.9% NaCl solution.

### Microbial Reduction of Hematite

The harvested cell suspensions were statically cultured in a minimal salts medium (DSM) with addition of 0.4 g L^-1^ natural and pure hematite (Supplementary Figure S1a), 10 mL vitamins solution, 10 mL mineral solution, and 10 mM piperazine-1,4-bisethanesulfonic acid (PIPES) per liter DSM. The natural hematite was isolated from an iron mine in Hunan province, central China. DSM contained (per liter): 5 g NaCl, 0.5218 g KCl, 0.7114 g NH_4_Cl, 0.1154 g MgCl_2_⋅6 H_2_O, 0.0504 g CaCl_2_⋅6 H_2_O, and 0.6786 g NaH_2_PO_4_. The medium was adjusted to pH 7 with 1 M NaOH, bubbled with oxygen-free N_2_, and then autoclaved. Filter-sterilized PIPES and 50 mM sodium lactate (final concentration 15 mM) were added after autoclaving. The vitamins and mineral solutions were added from stock solutions (Kieft et al., 1999). Soluble Fe(II) and Fe(III) were measured to determine the extent of hematite reduction using the ferrozine assay (Stookey, 1970). All microbial reduction experiments were designed in replicates, and data represent the mean of three parallel groups.

### Electrochemical Measurement of Cells Subjected to Different Treatments

Electrochemical measurements were operated on an auto-lab electrochemical workstation (CHI 1000C, CHI760E; Chenhua, Shanghai, China) with a single chamber and three-electrode MFCs (Supplementary Figure S2a). The chamber contained a platinum-wire counter electrode, an Ag/AgCl reference electrode with saturated KCl (potential versus the normal hydrogen electrode, +0.198 V), and an electrically conductive glass (FTO) working electrode covered with hematite. For electrochemical experiments, the electrolyte consisted of DSM solution from which all vitamins and minerals had been eliminated. All experiments were conducted under a nitrogen atmosphere by bubbling the solution with high-purity N_2_. The working electrodes were covered with hematite in accordance with the following procedure: (1) the FTO conductive glass (10 × 20 mm) was washed with acetone, ethanol, and deionized water, respectively, for 30 min in ultrasonic condition; (2) the electrode was air-dried; and (3) an approximately 1.0 cm^2^ area of the conductive side of the electrode was covered two times with 20 μL of the sonicated dispersion mixture (Supplementary Figure S2b). The mixture contained 400 μL anhydrous ethanol, 50 mg hematite powder (about 400 mesh), and 10 μL Nafion solution.

Enzymatic-treatment was carried out as follows: harvested cells (7 mg wet cell mL^-1^) were incubated with proteinase K (pK) from *Tritirachium album* (P2308; Sigma-Aldrich) at 30°C for 5 h. The final working concentration of pK was 500 μg mL^-1^ in sodium acetate buffer (10 mM, pH 7.2) (Randrianjatovo-Gbalou et al., 2017). Then, the cells were harvested by centrifugalization (6,000 rpm, 10 min), washed three times with 0.9% (wt vol^-1^) NaCl, and stored at 4°C for further use. The treated cells were marked as MR-1+pK. Cells were treated also with deoxyribonuclease (DNAse) from bovine pancreas (D5025; Sigma-Aldrich) at a final working concentration of 100 μg mL^-1^ in sodium acetate buffer (10 mM, pH 7.2) at 30°C for 5 h, and were named MR-1+DNAse. These cells were used to investigate the effect of EPS, including exopolysaccharides, exoprotein, and extracellular DNA, on EET processes.

Electrochemical parameters were measured as follows. For the cyclic voltammetry curve (CV), resting time was 8 s and scan rate was 10 mV s^-1^ at *E*_i_ = -0.6 V and *E*_f_ = 0.6 V. For differential pulse voltammetry (DPV), values were *E*_i_ = -0.6 V, *E*_f_ = 0.4 V, amplitude = 60 mV, pulse width = 200 ms, and potential increment = 6 mV. For I-t chronoamperometry, *E* was 0.3 V. To measure the electrochemical impedance spectrum (EIS), the prepared cell suspension (adjusted to about 1 × 10^7^ cells mixed with 1 mL of 1% Nafion aqueous solution) was drop-cast on the glassy carbon electrode to form a layer of cells and PIPES solution (50 mM, pH 7.0) was used as electrolyte. Nyquist plots, charge diffusion in solution (**R_s_**), and faradaic, impedance (**R_ct_**) were calculated by ZsimDemo 3.0 software (Eggleston et al., 2006; Logroño et al., 2017).

All electrochemical experiments were carried out in three or four replicates, and representative results are presented.

### Morphological Characterization of Cells, Reacted Hematite, and EPS

For three-dimensional fluorescence excitation-emission matrix (EEM) analysis of cell suspensions and EPS fractions, EEM spectra were recorded using a molecular fluorescence spectrometer (F-7000; Hitachi, Tokyo, Japan). EEM data were used to simulate complexing behaviors (Zhang et al., 2010). Samples were added to a 3-mL cuvette to measure fluorescence intensity at excitation wavelengths between 200–500 nm and emission wavelengths of 200–600 nm with a step of 5 nm.

For atomic force microscopy (AFM) imaging, 10-μL cell suspensions were dropped on glass slides with a diameter of 14 mm (Micro Cover Glass; CITOTEST, Nantong, China) and dried for 10 h under air flow. The slides were imaged by air-contact mode AFM (MultiMode 8; Bruker, Billerica, MA, United States) and data were analyzed with NanoScope Analysis software (Bruker).

The working electrode was observed on a Zeiss Supra 55 field emission scanning electron microscope (FE-SEM) (Carl Zeiss, Oberkochen, Germany) equipped with an energy dispersive spectrometer (EDS) (AZtecOne X-Max 150; Oxford Instruments, Abingdon, United Kingdom). An acceleration voltage of 15 kV was used. MR-1 cells on the working electrode were collected and suspended in a 2% formaldehyde solution (prepared in 10 mM phosphate buffer) overnight for cell fixation, and then dehydrated using a series of ethanol solutions (25, 50, 75, 95, and 100%). Finally, the cells were dried under ambient conditions. Following hematite reduction over a period of 7 days, 15 μL solution was dropped on a carbon-coated copper grid and then dried under anaerobic conditions. Prior to SEM observation, the samples were coated with platinum using a JEOL JFC-1600 auto fine coater device (JEOL, Tokyo, Japan).

To determine cell viability *in situ*, cells were stained with the LIVE/DEAD stain (Molecular Probes, Eugene, OR, United States) and analyzed with fluorescence microplate readers and a fluorescence microscope. To evaluate cell viability after EPS extraction and enzyme treatments, cells were stained also with the LIVE/DEAD BacLight Bacterial Viability Kit, containing two fluorescent dyes: SYTO 9 and propidium iodide. The staining procedure was conducted according to the manufacturer’s instructions. Cells stained with the two fluorescent dyes were observed under an inverted confocal fluorescence microscope (Zeiss 710). Fluorescence intensity was analyzed with ImageJ software^1^.

For scanning transmission X-ray microscopy (STXM) analysis, 15 μL of an experimental solution obtained after 10 days of microbial hematite reduction was dropped on a carbon-coated copper grid and then dried under anaerobic conditions. STXM analysis was conducted at the beamline 08U1A of Shanghai Synchrotron Radiation Facility (SSRF), at 3.5 GeV and a constant current of 230 mA. The copper grid was mounted on a sample holder and put into the STXM chamber. The distribution map of iron was acquired by digital division of two absorption-contrast images at dual-photon energies of *E*_1_ = 705.0 eV, *E*_2_ = 711.0 eV, and *E*_1_ = 709.0 eV (L3-edge). Spatial resolution was 30 nm and the flux was > 109 phs/s. An area of interest was scanned from 705.0 to 730.0 eV with an energy increment of 0.2 eV to measure the iron NEXAFS spectrum of each spot (30 × 30 nm) (Zhang et al., 2010).

For mineral identification of hematite and the hematite-covered working electrode, hematite powder (<200 mesh) was prepared by grinding and sieving, and the hematite-covered working electrode was dried directly in the air prior to measurement. Mineral components’ data were collected using the ARL X’TRA XRD system (Thermo Electron, Écublens, Switzerland) and a JY HR800 Raman spectroscope (HORIBA Jobin Yvon, Longjumeau, France).

## Results and Discussion

### EPS Are Efficiently Removed From MR-1 Cells

**FIGURE 1:**
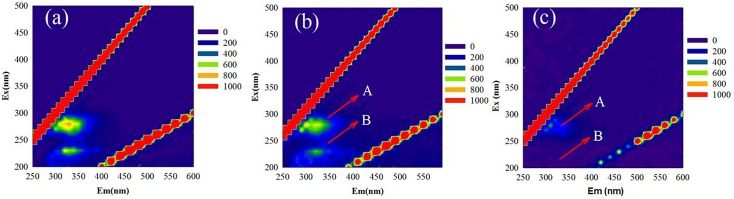
Typical three-dimensional fluorescence spectra of MR-1 cells at pH 6.0 with EPS **(a)** and without EPS **(b)**, and the spectra of isolated EPS from MR-1 cell **(c)**. (Excitation wavelengths of 200–500 nm and emission wavelengths of 200–600 nm, the wavelength step was 5 nm).

**FIGURE 2:**
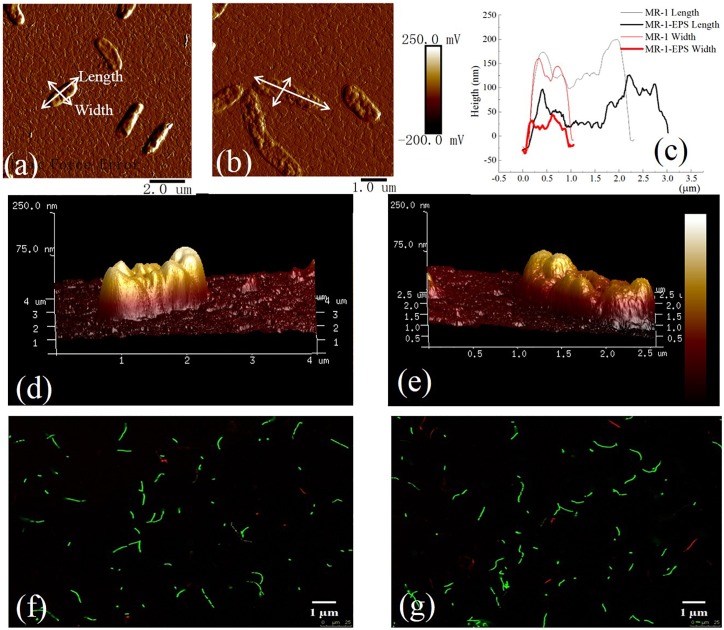
AFM images of MR-1 cell with EPS **(a,d)** and without EPS **(b,e)**. Height of cells in length and width for MR-1 cell with EPS and without EPS **(c)** [Text with white leader in **(a,b)**]. LIVE/DEAD staining of MR-1cells with EPS **(f)** and without EPS **(g)**. Excitation wavelength of 488 nm and an emission wavelength of 503 nm for SYTO 9 (green) and at 543 nm excitation collected at 605 nm for propidium iodide (red) on the 40x objective lens. The cell viability was detected by staining with SYTO 9 (green) (excitation/emission: 488/503 nm) and propidium iodide (red) (excitation/emission: 543/605 nm).

MR-1 cells with and without EPS were characterized by collecting EEM spectra. The signals at excitation wavelengths of 220–230 nm (peak A) and 270–280 nm (peak B) (Figure 1) were assigned to protein-like fluorescence (Chen et al., 2003). The EEM spectra of regular MR-1 cells presented two maxima of 828.7 (peak A) and 686.0 (peak B), which became 743.9 and 531.8, respectively, in MR-1-EPS cells. AFM images (Figure 2) revealed that MR-1 cells with EPS (Figure 2a,d) were much larger than those without EPS (Figure 2b,e) both in length and width. Based on the size distribution of the two cell types, the thickness of the removed EPS layer was calculated to be 61 ± 17.32 nm (Table 1 and Supplementary Figure S3). LIVE/DEAD staining indicated that removal of EPS had little effect on cell viability (Figure 2f,g).

**Table 1:** Surface physicochemical properties of cells with different treatment (“-” means undetected).

	Zeta potential (mV)	Mean value size (nm)	TOC (mg g^-1^ wet cell)
MR-1	–9.07 + 0.10	932.00 ± 24.43	–
MR-1-EPS	–9.36 + 0.42	871.67 ± 10.21	–
MR-1+pK	–10.13 + 0.81	921.03 ± 11.56	–
MR-1+DNAse	–13.14 + 0.94	965.21 ± 33.01	–
EPS	–6.73 + 0.04	–	1.06 ± 0.01

	**Exopolysaccharides (mg g^-1^ wet cell)**		**Exoproteins (mg g^-1^ wet cell)**

MR-1	5.12 ± 0.56		2.93 ± 0.16
MR-1-EPS	3.71 ± 0.22		2.62 ± 0.33
MR-1+pK	5.13 ± 0.20		1.17 ± 1.07
MR-1+DNAse	4.88 ± 0.23		2.03 ± 0.10
EPS	0.87 ± 0.08		0.45 ± 0.06


The zeta potential of MR-1 and MR-1-EPS cells at pH 7.0 was -9.07 mV and -9.36 mV, respectively. Thus, the surface of MR-1-EPS cells had a more negative potential. The TOC of removable EPS in MR-1 cells was 1.06 ± 0.01 mg g^-1^ wet cells, and the EPS contained 0.45 ± 0.06 mg exoproteins and 0.87 ± 0.08 mg exopolysaccharides per gram of wet MR-1 cells. The pH of the EPS solution was 6.8, and the EPS produced a potential difference of -6.73 mV (Table 1) at pH 7.

### EET Efficiency Based on Macroscopic Microbial Reduction of Hematite

**FIGURE 3:**
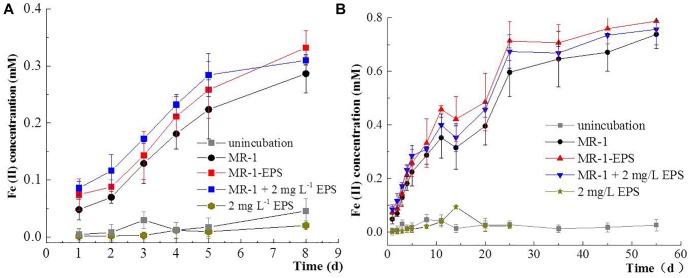
Concentration changes of Fe(II) in the first 7 days **(A)**, and Concentration changes of Fe(II) from 1 to 55 days **(B)** for microbial reduction of Hematite.

A series of batch experiments on microbial hematite reduction were performed by employing different MR-1 cells, including untreated MR-1, MR-1-EPS, and MR-1+EPS (MR-1-EPS with EPS addition, final concentration 2 mg L^-1^). As shown by Fe(II) concentration changes, the extent of hematite reduction followed the order: (MR-1 + EPS) > (MR-1-EPS) > (MR-1) in the first 5 days (Figure 3A), and (MR-1-EPS) > (MR-1 + EPS) > (MR-1) after 5 days (Figure 3B). Both MR-1 + EPS and MR-1-EPS cells strongly reduced insoluble hematite, but EPS alone could not reduce the insoluble hematite, which is comparable with the reduction of soluble U(VI) or Cr(VI) (Harper et al., 2008; Cao et al., 2011; Harish et al., 2012). Fe(II) concentration was unexpectedly 20–30% higher in MR-1-EPS cells, which were regarded as unable of carrying out EET processes between the outer membrane and hematite.

**FIGURE 4:**
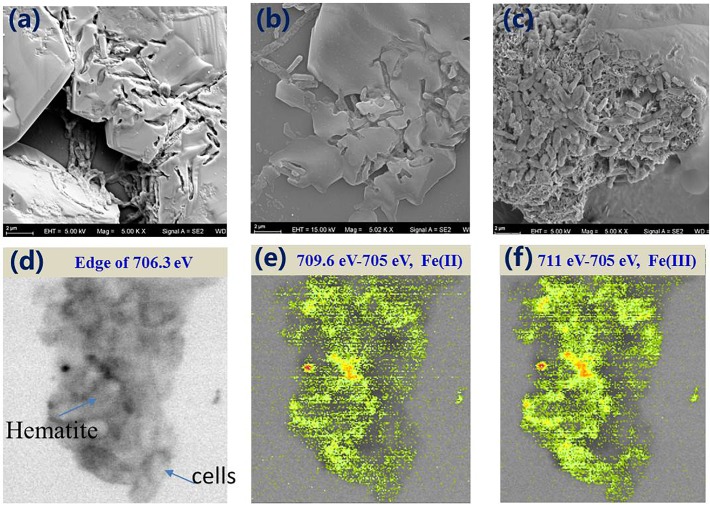
SEM images of MR-1-EPS **(a)**, MR-1 **(b)**, and MR-1 + EPS **(c)** groups after 7 days hematite microbial reduction. The STXM images of MR-1 + EPS group after 7 days reaction at 706.3 eV **(d)**, distribution of Fe(II) species (709.6 eV–705 eV) **(e)**, and Fe(III) species (711 eV–705 eV) **(f)** (image size is 6.5 **×** 6.0 μm).

Our observation suggests that EPS exerted a negative influence on microbial reduction of minerals, contrasting with their positive aggregation effect during biofilm formation (Flemming et al., 2007). The direct physical contact between outer membrane terminal enzyme and hematite was severed as more than 54–56% of Fe(III) reductase was localized in the outer membrane (Smith et al., 2013). SEM images confirmed the morphological differences between experimental groups (Figure 4a–c). In the MR-1-EPS group, etching pits with cell-like shape were visible on the hematite surface after 7 days, whereas no obvious etching pits were observed in the MR-1 group. This finding further supports the importance of direct contact between terminal reductases and iron oxide surfaces in promoting solid mineral reduction, and is in agreement with previous studies (Liu et al., 2016).

In contrast, in the MR-1+EPS group, more cells were found to aggregate together, allowing the biofilm to develop, which is consistent with this group’s higher Fe(II) concentration. STXM analysis confirmed that Fe(II) distributed evenly on the surface of MR-1 cells, aggregating closely with hematite particles, as seen in the MR-1+EPS group (Figure 4d–f).

### Current Density Output of Different Cells in MFCs

**FIGURE 5:**
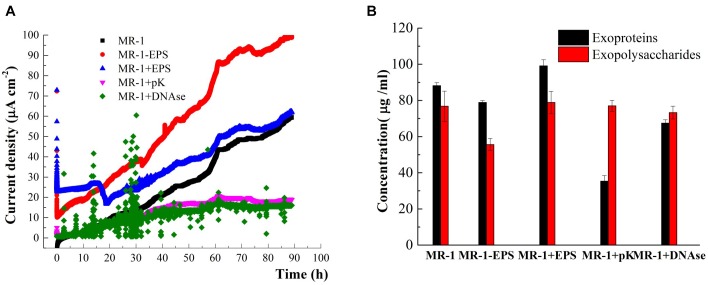
Time-current generation in MFCs harbored different cells **(A)** (cells were incubated in the DSM added 15 mM lactate and 50 mM PIPES buffer solution, pH = 7.0). The content of exoproteins and exopolysaccharides before and after or PK or DNase enzyme treatments and EPS removal **(B)**.

The EET capabilities of MR-1 and MR-1-EPS cells during hematite reduction were examined further using electrochemical methods. The I-t curves of the single three-electrode chamber (Figure 5A) indicated that MR-1-EPS and untreated MR-1 cells on the anode could produce electric current continuously for at least 90 h. Furthermore, MR-1-EPS cells produced 2–3 times more current than MR-1 cells. For example, at 40 h, the current density of MR-1-EPS cells was 56 μA cm^-2^ whereas that of MR-1 cells was only 15 μA cm^-2^. Thus, the removal of EPS remarkably enhanced EET processes on the interface of the working electrode. Given that the proteins with the highest redox activity distribute preferentially near the outer membrane, we think that direct contact between the outer membrane of attached cells and the hematite-covered electrode was responsible for the enhanced EET (Lawrence et al., 2016). Notably, during the initial 15 h, the current density of MR-1+EPS cells was higher than that of MR-1-EPS cells, and was similar to the experimentally observed microbial reduction of hematite (Figure 3). This phenomenon could be attributed to increased amounts of conductive proteins and electron shuttles on the electrode due to EPS addition. However, at 18 h, the current density clearly decreased, possibly as a result of weaker mechanical stability and adhesion of EPS, which then led to the dispersion of resident cells (Hobley et al., 2015).

The molecular mechanisms and essential roles of typical proteins in the EET, such as c-type cytochromes and microbial nanowires, have been proved recently by Shi and colleagues (Shi et al., 2012, 2016). To determine the contribution of EPS components to EET processes between cells and hematite, cell-impermeable pK and DNAse were used to hydrolyze exoproteins and extracellular DNA *in situ* (Randrianjatovo-Gbalou et al., 2017). The treatments caused little damage to cells (Supplementary Figure S4). After pK treatment, 60% of initial exoproteins were digested, whereas DNAse was able to remove 25% of the initial exoproteins mainly through their reduction. In contrast, significant removal of exopolysaccharides was not observed (Figure 5B), in agreement with previous studies (Xu et al., 2011; Lawrence et al., 2016). pK and DNAse treatments remarkably decreased current density compared with that of normal MR-1 cells (Figure 5A, purple and green lines). Therefore, digestion of proteins in the EPS layer likely terminated the electron transfer pathways. Moreover, as one electron can only hop over a distance of 1–2 nm (Yong et al., 2017), the proteins removed by DNAse treatment must have been very close to EET mediators. Finally, these findings reveal that the exopolysaccharides left in the EPS hardly contributed to EET processes, and current density dropped almost to blank (Figure 5A, purple and green curves). Previous studies have reported that MR-1 mutants deficient in the polysaccharide synthesis-associated gene SO3177 generated 50% more current density than the wild-type strain (Kouzuma et al., 2010). Nevertheless, it is worth noting that polysaccharides can affect electron transfer by increasing cell sensing as well as performance in bioreactors (Hamm et al., 2003; Keerthi et al., 2018).

### Electrochemical Characteristics of Treated MR-1 Cells on the Electrode

**FIGURE 6:**
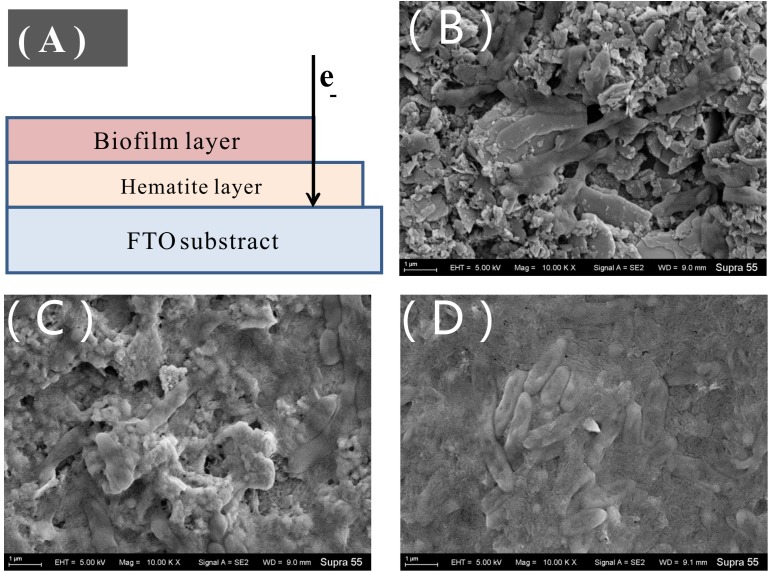
Sketch of thin biofilm on hematite/FTO electrode **(A)**, SEM images of the biofilm on the hematite/FTO electrodes of MR-1-EPS **(B)**, MR-1 **(C)**, and MR-1 + EPS **(D)** groups after 72 h at the constant voltage 0.3 V.

Different redox signals on the contact interface can be readily recorded using the working electrode (Cheng et al., 2006; Costa et al., 2018). MR-1-EPS, MR-1, and MR-1+EPS cells attached to the hematite/FTO working electrode were seen to form a thin biofilm on the electrode (Figure 6A) at a constant potential of -0.3 V. No new mineral phase could be detected by XRD analysis (Supplementary Figure S1b), suggesting no evidence for redox reactions involving hematite on the electrodes. The redox characteristics on the interface of the thin biofilm were assessed by CV, DPV, and impedance curves.

**FIGURE 7:**
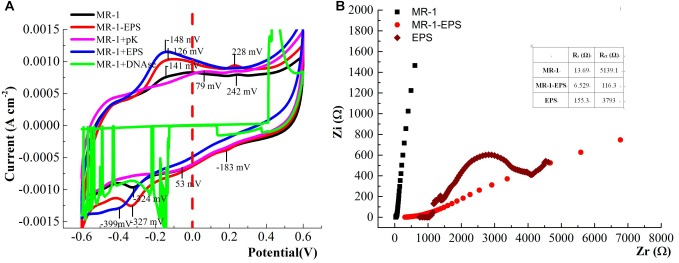
CV curves of the biofilm colonized electrodes. after 24 h for MFCs harbored different cells (Test condition: DSM medium added 15 mM lactate and 50 mM PIPES buffer solution at 0.3 V potential, pH = 7.0, scan rate: 60 mV S-1) **(A)**. The Nyquist curves of the electrode treated with different cells (frequency range: 10^-2^–10^5^ Hz, Zr and Zi was the real part and virtual part of Resistance, respectively) **(B)**.

As shown in CV curves (Figure 7), a pair of well-defined redox peaks versus Ag/AgCl were detected at -0.238 V (cathodic, Epc) and -0.019 V (anodic, Epa) in the MR-1-EPS group. The midpoint potential (*E*_0_) was calculated as -0.128 V based on *E*_0_ = (Epc + Epa)/2. While the *E*_0_ of the MR-1 group was -0.138 V, the left shift of E_0_ demonstrates that EPS removal decreased the redox potential relative to that of regular MR-1 cells and promoted EET. In addition, there were no obvious redox peaks in MR-1 cells except at -0.324 V versus -0.141 V, which was attributed to flavins (Lin et al., 2018). A well-defined redox pair at -0.126 V (anodic) and -0.183 V (cathodic) was caused by the outer membrane c-type cytochromes (Gorby et al., 2006), and a pair of undefined peaks at -0.126/-0.327 V and 0.228/0.053 V was detected in MR-1-EPS cells (Figure 7, red line and Supplementary Figure S5, red line). The latter two peaks could be assigned to other outer membrane proteins or protein-like substances combined with an EEM absorbance peak at 230/330 nm (Figure 1) (Holbrook et al., 2006; Priester et al., 2006).

More redox pairs (-0.126/-0.327 V and 0.228/0.053 V) were detected in the MR-1-EPS group. Given that the strong voltammetry redox signals assigned to cytochromes could be further potentiated by EPS depletion and flanked by additional signals derived from flavins (Yong et al., 2017), our results suggest that more outer membrane proteins were engaged in interface processes. This is consistent with the relatively high hematite-reducing performance of MR-1-EPS cells. Moreover, this observation reinforces the importance of enabling physical contact between the terminal reductases (Liu et al., 2016). The disappearance of redox peaks in the MR-1+pK and MR-1+DNAse groups indicates extremely low current output (Figure 5A, purple and green curves), and could be explained by the lack of redox proteins for EET.

SEM images revealed that MR-1-EPS cells covered the electrode independently of each other (Figure 6B). The presence of EPS may determine the mode of hematite-cell contact: MR-1-EPS cells were in direct contact with hematite, whereas other cells aggregated together and were spatially separated from hematite by EPS (Figure 6C,D).

The redox potential peaks almost disappeared at a lower scanning rate of 10 mV S^-1^ (Supplementary Figure S6), suggesting that the reactions on the electrode were irreversible, and the charge/electron transfer was a potential difference-driven process rather than a concentration-driven diffusion event (Qian et al., 2014; Logroño et al., 2017). The interface resistance of different working electrodes was detected and simulated by Nyquist analysis. The faradaic resistance **R_s_** of EPS was clearly higher than those of MR-1 cells with and without EPS (Figure 7B and Table 1). This observation may be attributed to the inability of exoproteins or electron shuttles in isolated EPS to form an intact electron transfer chain and then absorb ions from solution (Villacorte, 2016). Based on the estimate of the semicircle diameter, the **R_ct_** of the MR-1-EPS/FTO electrode was 116.3 Ω, which was 44.2 times lower than that of the MR-1/FTO electrode, 5,139.1 Ω (Table 1). The lower **R_ct_** indicates a faster electron transfer for MR-1-EPS cells. In general, EET processes involve mainly diffusion and charge transfer (Williams and Scherer, 2004; Zill et al., 2011). Existing studies have rarely focused on EET process in the absence of an EPS layer. Exopolysaccharides left in the EPS hardly contributed to EET processes (Figure 5A); simulation from the EIS curve further indicated that the EPS matrix deactivated the EET process and contributed to a greater **R_ct_**.

**FIGURE 8:**
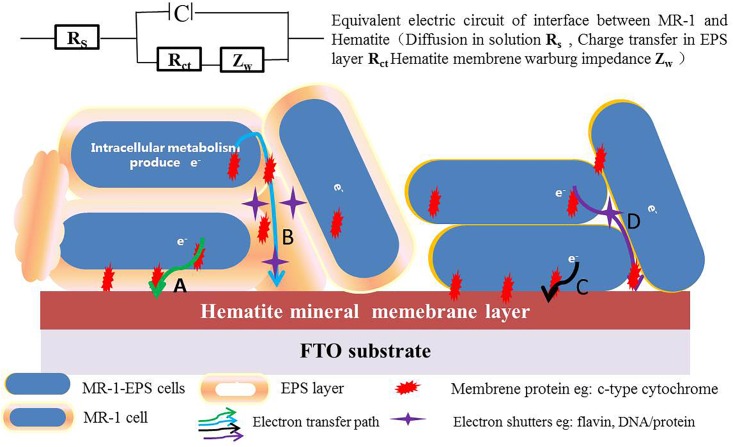
The proposed EET pathways in absence and presence of EPS (**Path A**: electrons are transported via redox shuttles or proteins on the outer-membrane and cross the mono EPS layer through direct contact, and **Path B**: electrons hop across multi-EPS layer and arrive at hematite surface with the electron shutters in presence of EPS. **Path C**: electrons directly hop to reactive surface sites of hematite, and **Path D**: electrons are transported via various shuttles in absence of EPS).

Based on the experimental results and electrochemical characterization of different MR-1 cells, we propose a two-pathway mechanism for mediating EET across the EPS layer. (1) For regular cells, electron transport depends on redox shuttles or outer membrane redox proteins, such as c-type cytochrome, flavin/c-type cytochrome compounds, redox proteins, or DNA-containing redox proteins. These redox shuttles may take the electrons either directly across the mono-EPS layer (path A), or they may act in concert with other proteins to allow electrons to hop across the multi-EPS layer (path B) before reaching the hematite surface. (2) For cells without EPS, electrons are carried by outer membrane redox proteins, which transfer them directly to the hematite surface when physical contact is possible (path C), or they are transported via various shuttles without crossing the multi-EPS layer (path D) (Figure 8).

### Roles of EPS in EET Processes Between MR-1 Cells and Hematite

Microorganisms have been thought to transfer electrons between cytoplasmic membranes and extracellular insoluble minerals through a network of structural proteins (Shi et al., 2016; Keogh et al., 2018). Outer membrane proteins commonly present high redox activity (Lawrence et al., 2016), they are also crucial and selective for EET (Shi et al., 2007; Sheng et al., 2016). Electron “hopping” is the most likely mechanism for electron transfer across the EPS layer (Yong et al., 2017). The EPS matrix constitutes the framework of microbial biofilms, and thus enables direct physical contact between the cell and mineral surfaces (Singh et al., 2006; Decho, 2010). This study indicates that EPS removal favors the exposure of redox proteins on the outer membrane to redox active sites on hematite’s surface. This, in turn, promotes the EET process, as indicated by higher hematite reduction and greater current density output (Figure 3, 5). At the same time, extracellular proteins and associated DNAs within the EPS layer are critical for the EET process, as the digestion of proteins and DNAs evidently terminated the EET chain.

Almost all microbial cells are surrounded by EPS, which likely supported the first, wide distribution of life on earth, through what is now known as “The perfect slime” (Hay, 1981; Flemming, 2016). However, the diffusion of ions is substantially slower across the EPS matrix than in solution (Kornyshev et al., 2003), which is consistent with the dramatically high R_s_ of EPS (Table 1). Moreover, the charge transfer resistance **R_ct_** of the MR-1/hematite electrode was also substantially higher than that of the MR-1-EPS/hematite couple (Figure 6B and Supplementary Table S1). By and large, the EPS matrix layer deactivated EET due to a higher content of non-conducting exopolysaccharides.

EPS are crucial for the development of biofilms, providing fundamental cohesive force for the colonization of solid surfaces (Liu et al., 2018). Our findings suggest that exopolysaccharides of the EPS layer substantially inhibit EET processes by increasing charge transfer resistance. The latter is essential also for electron “hopping” across the EPS layer. Removal of EPS increases the exposure of redox active sites to physical contact. Because EPS secretion and biofilm development can be regulated by several genes, such as SO3177 (Saltikov and Newman, 2003) and bifA (Zheng et al., 2018), genetic manipulation can dictate the balance between EET efficiency and the structural and protective role of biofilms.

## Conclusion

The EPS matrix plays a fundamental role in allowing cells to stay in each other’s proximity for extended interaction, energy capture, mechanical strength, and other properties. EPS can preliminarily enhance bacterial cell-mineral surface contacts and favor interface reactions, as observed here in regular cells and those subjected to EPS addition. Exoproteins and electron shuttles mediate electron transfer. Both the enhanced microbial hematite reduction and higher current density outputs observed in electrochemical measurements indicate that removal of EPS from *Shewanella oneidensis* MR-1 cells increases the exposure of outer membrane redox proteins to the hematite surface and promotes EET efficiency. EET performance decreased dramatically as the spatial redox mediators were digested *in situ*. The inhibitory effect of EPS polysaccharides on electron transfer was clearly demonstrated, and the exopolysaccharides acted as non-conductive substances during microbial reduction. Over the past decades, the quantity and composition of EPS have been shown to be controllable by genetic engineering techniques (Kouzuma et al., 2010) and culture medium conditions (Liang et al., 2011). As removal of EPS increases the exposure of redox active sites on bacterial cells and related surface interactions, we suggest regulating the balance between EET efficiency, biofilm development, and the natural protective function of bacterial cells to control microbe-mineral interactions.

## Author Contributions

LG and XL designed the experiments. LG carried out the experiments. HL, JL, and WL assisted with experiments of microbial reduction of hematite. LG analyzed the experimental results. HL analyzed the STXM data. RS and JZ assisted with the analysis of electrochemical data including CV, DPV, and EIS curves. LG wrote the manuscript. XL, DZ, and RW helped to revise the manuscript.

## Conflict of Interest Statement

The authors declare that the research was conducted in the absence of any commercial or financial relationships that could be construed as a potential conflict of interest.

## Footnotes

1 https://imagej.nih.gov/ij/download.html
