# Supplementary figures and images for: Mediation of Extracellular Polymeric Substances in Microbial Reduction of Hematite by Shewanella oneidensis MR-1
Source: Front Microbiol. 2019 Mar 29;10:575. doi: 10.3389/fmicb.2019.00575 (PMC6449630; doi:10.3389/fmicb.2019.00575)

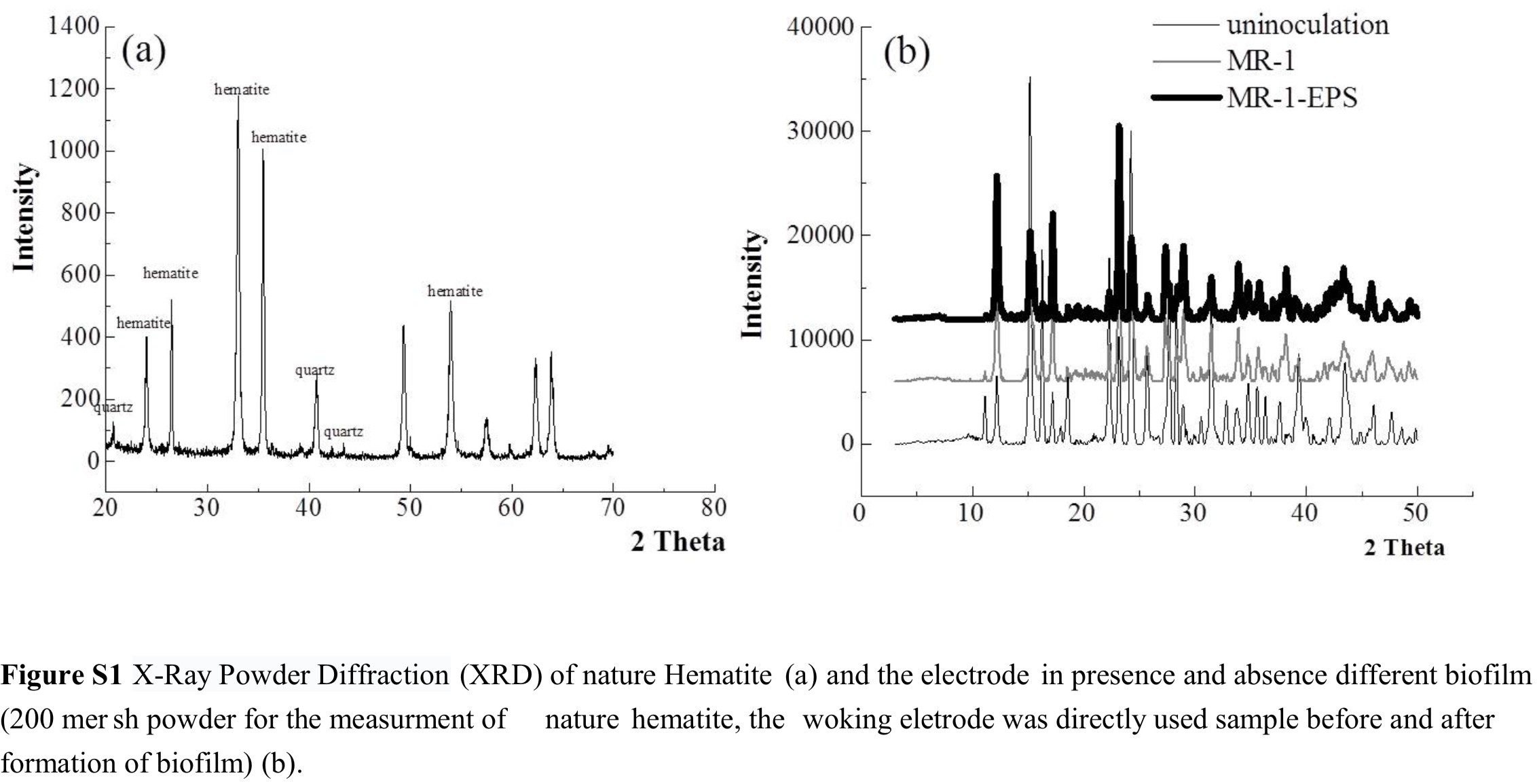

Supplement: Supplementary file 1 [file Image_1.JPEG]

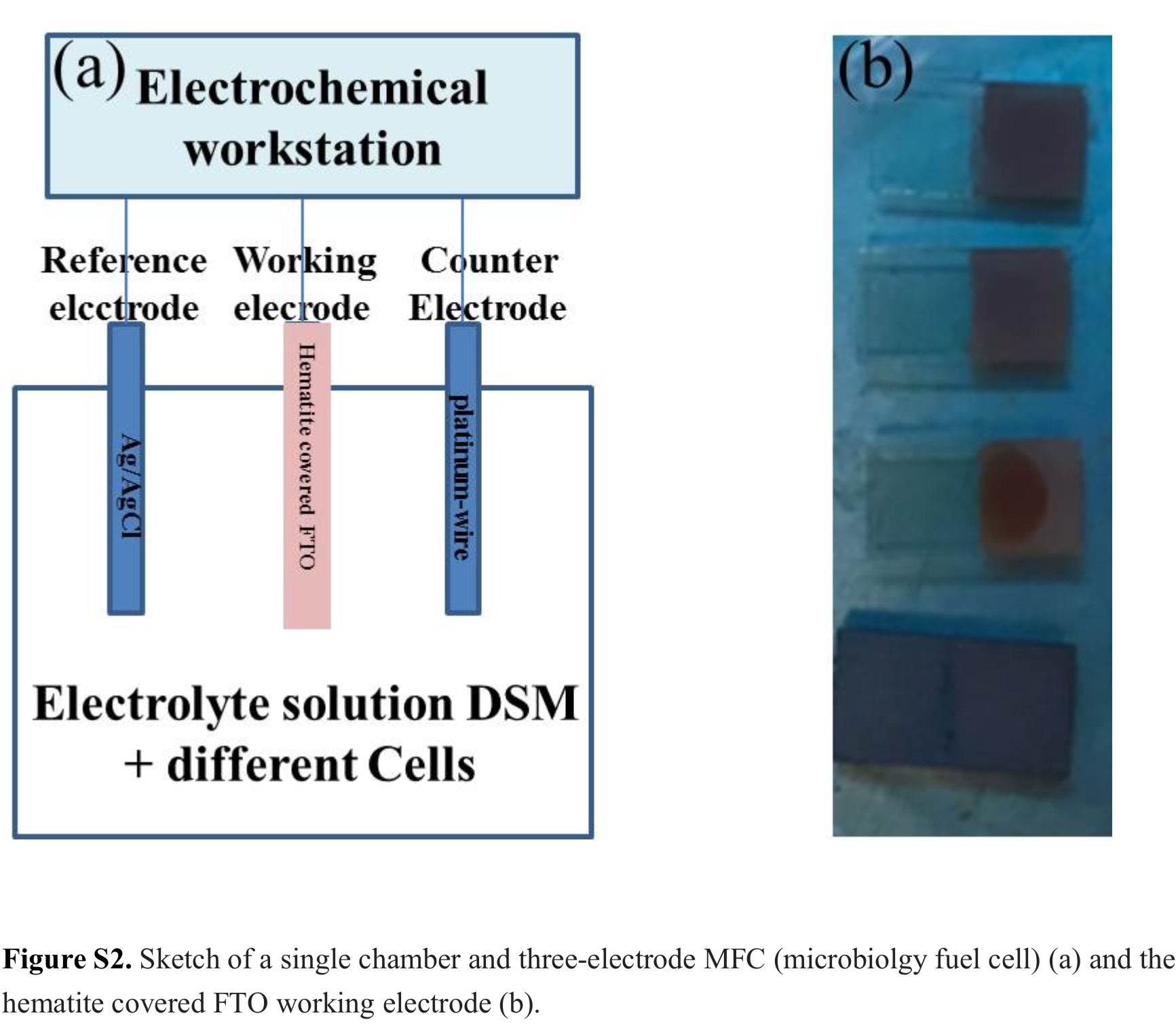

Supplement: Supplementary file 2 [file Image_2.JPEG]

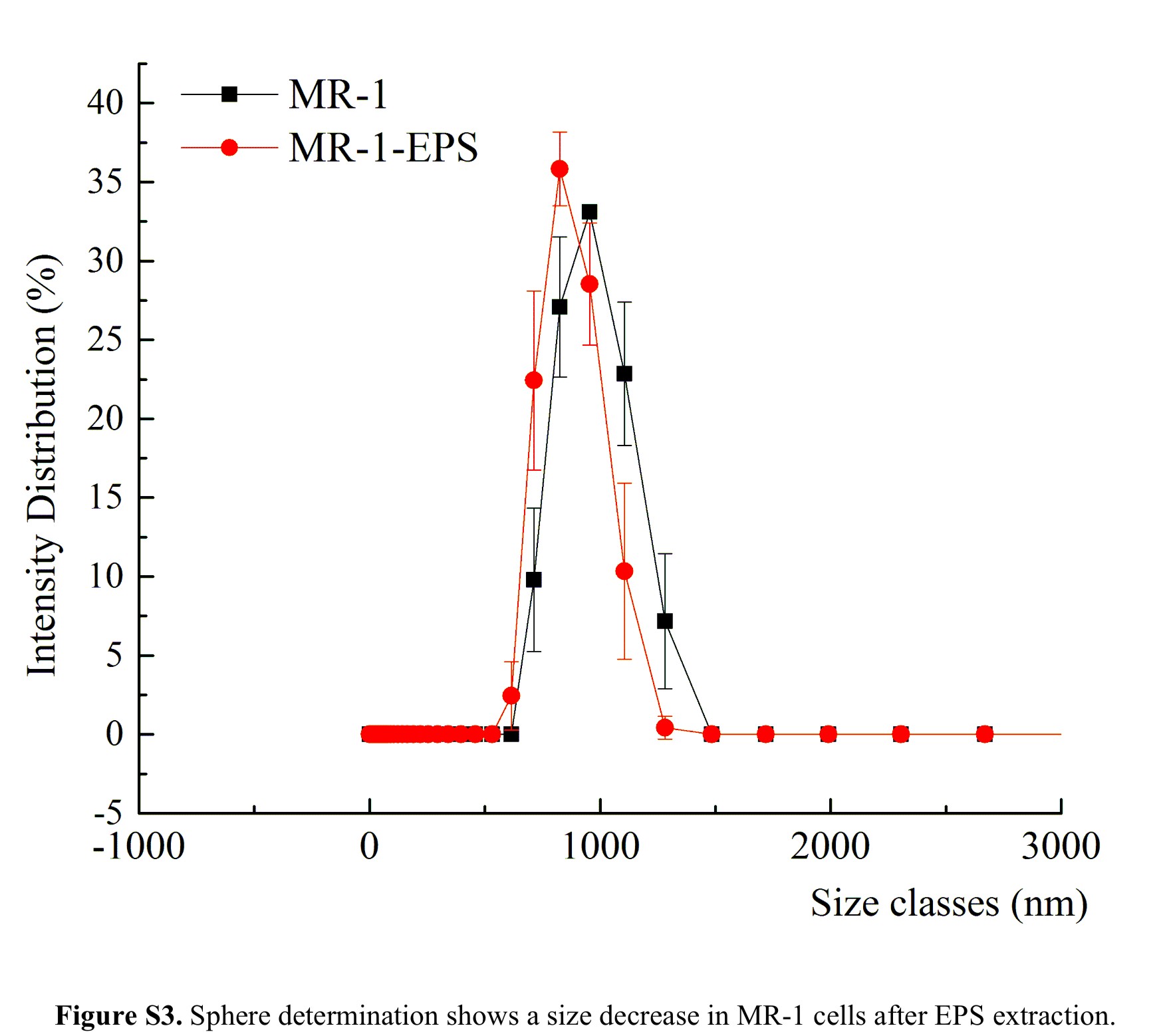

Supplement: Supplementary file 3 [file Image_3.JPEG]

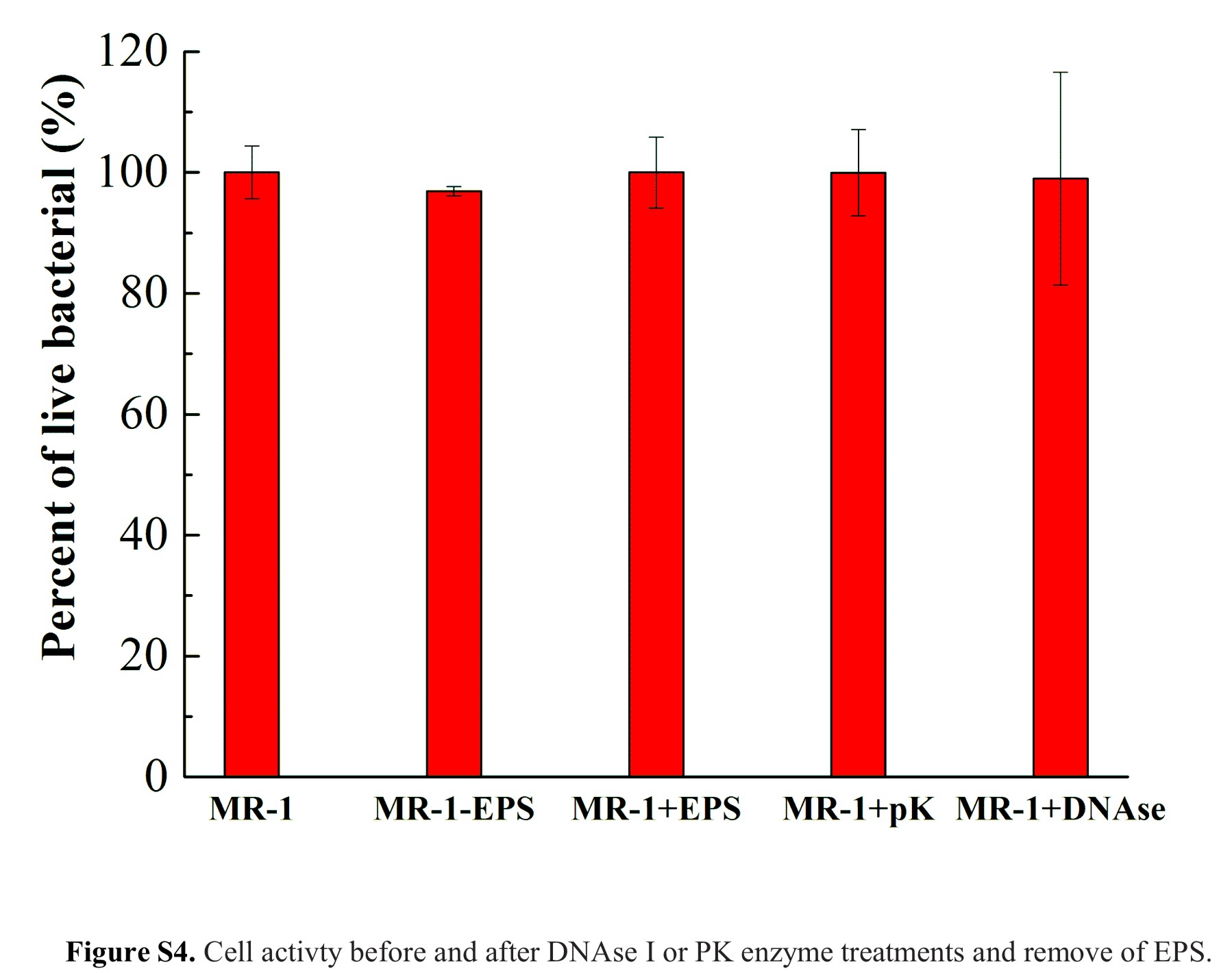

Supplement: Supplementary file 4 [file Image_4.JPEG]

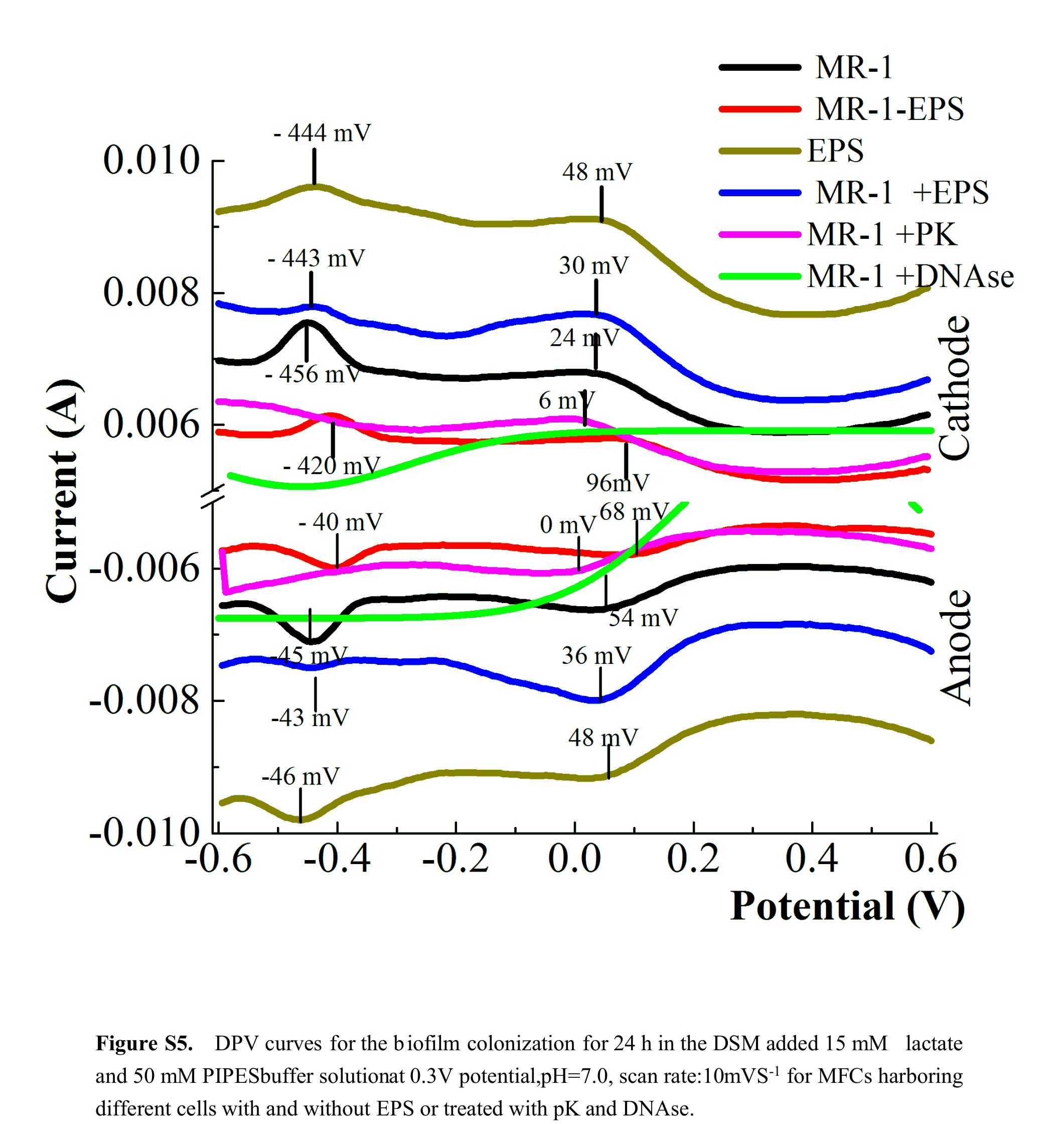

Supplement: Supplementary file 5 [file Image_5.JPEG]

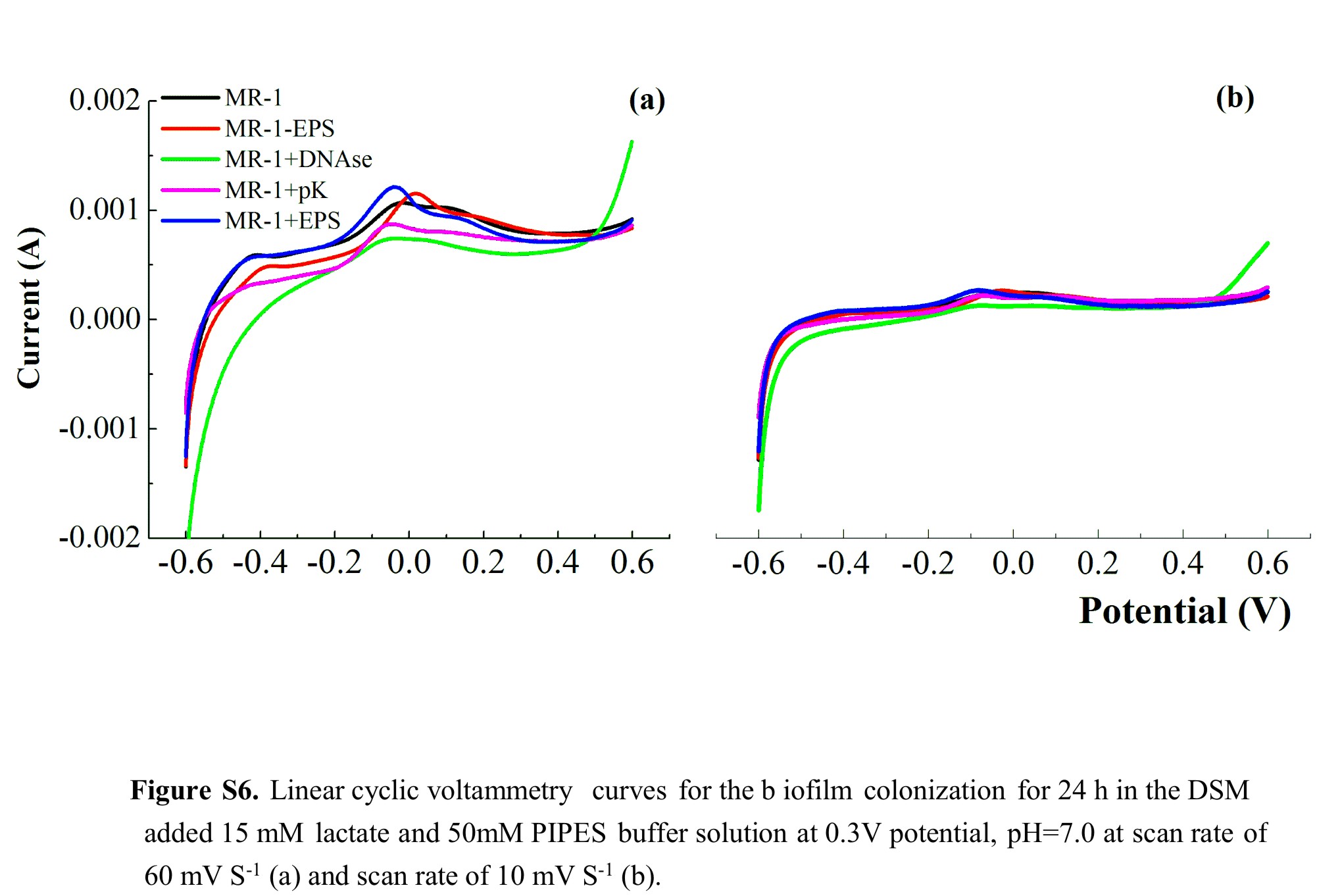

Supplement: Supplementary file 6 [file Image_6.jpeg]
