# Supplementary material for: Mediation of Extracellular Polymeric Substances in Microbial Reduction of Hematite by Shewanella oneidensis MR-1
Source: Front Microbiol. 2019 Mar 29;10:575. doi: 10.3389/fmicb.2019.00575 (PMC6449630; doi:10.3389/fmicb.2019.00575)
Supplement: Supplementary file 7 [file Table_1.docx]

**Table S1.** Impedance parameters for electrodes with different treatments

|  | **Opencicuit potential (mV)** | **R_s_ (Ω)** | **R_ct_ (Ω)** | **Z_w_ (Ω)** |
| --- | --- | --- | --- | --- |
| **MR-1** | -387.0 | 13.69 | 5139.1 | 0.005126 |
| **MR-1-EPS** | -428.8 | 6.529 | 116.3 | 0.00608 |
| **EPS** | -115.3 | 155.3 | 3793 | 0.0006526 |

0.01毫米=10微米
1微米--------12500目
1.3微米--------8000目
2微米--------6250目
2.6微米--------5000目
5微米--------2500目
6.5微米--------2000目
10微米--------1250目
15微米--------800目
20微米--------625目
33微米--------425目
37微米--------400目
44微米--------325目
74微米--------200目
149微米--------100目
350微米--------45目

ppm = mg/L

ppb = μg/L

ppt = ng/L
